# Cardiovascular Disease Risk in Rheumatoid Arthritis Anno 2022

**DOI:** 10.3390/jcm11102704

**Published:** 2022-05-11

**Authors:** Bas Dijkshoorn, Reinder Raadsen, Michael T. Nurmohamed

**Affiliations:** 1 Amsterdam Rheumatology and Immunology Center, Location Reade, Department of Rheumatology, Dr. Jan van Breemenstraat 2, 1056 AB Amsterdam, The Netherlands; r.raadsen@reade.nl (R.R.); m.nurmohamed@reade.nl (M.T.N.); 2 Department of Rheumatology, Amsterdam Rheumatology and Immunology Center, Amsterdam University Medical Centers, 1007 MB Amsterdam, The Netherlands

**Keywords:** rheumatoid arthritis, inflammation, cardiovascular diseases, cardiovascular risk management

## Abstract

The risk for developing cardiovascular diseases (CVD) in rheumatoid arthritis (RA) patients is 1.5 times higher compared to the general population. This risk is partly due to the contribution of systemic inflammation in increased atherogenesis, while an increased prevalence of “traditional” cardiovascular risk factors, such as hypertension and dyslipidemia, is also attributed to nearly 50% of the total CVD risk. Most anti-rheumatic medication partly reduces this CVD risk, primarily by reducing inflammation. The increased risk is recognized by most guidelines, which advise consequent screening and multiplying calculated risk scores by 1.5. However, screening in daily clinical practice is poorly done, and RA patients often have undiagnosed and untreated risk factors. In conclusion, even nowadays, RA patients still have an increased risk of developing CVD. Advances in anti-inflammatory treatment partly mitigate this risk, but RA patients need mandatory screening for CV risk factors to turn their CVD risk towards that of the general population.

## 1. Introduction

Rheumatoid arthritis (RA) is the most prevalent chronic inflammatory joint disease, with a prevalence of 460 per 100,000 people [1]. RA is characterized by chronic and destructive synovitis, leading to irreversible joint damage. However, the systemic inflammation characteristic of the disease also causes problems beyond the joints, such as interstitial lung disease, vasculitis and cardiovascular disease (CVD). In this review, we focus on CVD, discussing the CVD risk, the impact of anti-rheumatic treatment on CVD and the current guidelines regarding cardiovascular risk management in patients with RA.

## 2. Etiology of Rheumatoid Arthritis and Atherosclerosis

The etiology of RA is still not completely understood; however, both genetic and environmental factors contribute to the development of the disease. Several genetic and epigenetic components have been linked to RA, along with several environmental factors such as cigarette smoke, dust exposure and our microbiome. Other environmental factors, such as hormones, might explain the higher risk in women. Progression of the disease often starts years before the onset of symptoms with the development of certain autoantibodies, such as rheumatoid factor (RF) and anti-citrullinated protein antibodies (ACPA). ACPA-positive RA patients have more severe disease activity and increased CV mortality, and the presence of these antibodies might also contribute to the atherosclerotic process. ACPA were also found in non-RA patients with CVD and were again associated with worse CVD outcomes [2].

After this initial, abnormal immune response has been established, which may take several years, large quantities of RF and ACPA are produced by plasma cells. These auto-antibodies subsequently activate macrophages through complement and Fc receptor binding, primarily in the joints. The local inflammation, enhanced by activated T cells, causes damage to the synovium, inducing pain, swelling and stiffness of the joints. However, the inflammatory reaction is not limited to the synovium but is systemic, occurring throughout the bloodstream. Several pro-inflammatory cytokines and chemokines, such as tumor necrosis factor-alpha (TNF-α) and interleukins 1 and 6 (IL-1/IL-6), are produced, which attract activated B and T cells, monocytes and macrophages. 

Recently, the JAK/STAT signaling pathway, which regulates cytokine signaling, has been found to play a role in the pathogenesis of RA [3]. This pathway is activated by IL-6, among other cytokines, and increases the production of pro-inflammatory cytokines [4]. This all leads to more systemic inflammation, causing extra-articular comorbidities, such as atherosclerosis [5].

The formation of atherosclerotic plaques is the main mechanism in the development of CVD in RA patients. The pathogenesis is multifactorial and has vascular, metabolic and inflammatory components [6]. Dyslipidemia, hypertension, smoking, and inflammation, among other risk factors, contribute to atherogenesis by causing damage to the endothelium of the arteries. Dysfunctional endothelial cells have increased permeability, enabling LDL to become trapped in the intima of the arterial walls, where it becomes oxidized by phospholipases and free radicals [7]. Increases in adhesion molecules on the endothelium cause monocytes to migrate to the intima, which subsequently differentiate into macrophages. Macrophages then ingest the accumulated lipids, turning into “foam cells”, and can microscopically be seen as a “fatty streak” in the arteries. The foam cells, along with damaged endothelial cells, produce several cytokines, such as TNF-α and IL-6, which attract and activate more leukocytes, endothelial and smooth muscle cells [8]. Some foam cells eventually die from apoptosis, creating a necrotic nucleus in the atherosclerotic plaque. Smooth muscle cells, macrophages and T-lymphocytes form a fibrous cap on the endothelial side of the plaque [9]. Atherosclerotic plaques can either gradually grow and cause chronic ischemia or become unstable and cause acute cardiovascular events [10].

It is widely accepted that chronic inflammation, such as in RA, can amplify the process of atherosclerosis [11]. The systemic inflammation causes endothelial dysfunction, leading to the earlier and faster progression of atherosclerotic plaques. Several of the underlying mechanisms are shared by RA and atherosclerosis [8]. TNF-α, one of the most important pro-inflammatory cytokines in RA, is also produced by foam cells and contributes to endothelial dysfunction. It also upregulates the expression of adhesion molecules, resulting in the accumulation of more immune cells in the plaques. IL-6 promotes fatty streak development, and IL-1 plays a role in the regulation of macrophages and T-helper 17 cells.

In addition to directly influencing the development of atherosclerotic plaques, systemic inflammation also enhances the CV risk factors. By contributing to endothelial dysfunction and increased oxidative stress, inflammation leads to increased systemic vascular resistance and, thus, to hypertension [12]. Inflammation in RA also leads to the increased catabolism of lipoproteins, often resulting in a decrease in the HDL and LDL levels while still associated with the future CVD risk in what is called the “lipid paradox”. Furthermore, inflammation causes the HDL levels to become pro-atherogenic by reducing its cholesterol efflux capacity, the ability to accept cholesterol from macrophages, which is an independent risk factor for CVD [13,14]. Higher levels of lipoprotein(a), a pro-inflammatory and pro-atherogenic modified LDL particle, are also associated with RA and are an independent risk factor for CVD [15]. Treatment with anti-inflammatory medication largely reverses these effects, with a subsequent CVD risk reduction in RA patients.

## 3. Cardiovascular Risk

It has become increasingly known that CVD is a common comorbidity in RA patients, resulting in a more severe disease burden. The risk of developing CVD in RA patients is 1.5 times higher compared to the general population [16,17]. A very recent cohort study demonstrated that, even nowadays, this risk still approaches that of diabetes patients [18]. Several meta-analyses have indicated an increased chance of CV death by 50–60% [19,20]. This risk is partly due to the contribution of systemic inflammation to increased atherogenesis, while the “traditional” CV risk factors are also attributed to nearly 50% of the total CVD risk [21].

Several of these risk factors have increased the prevalence in patients with RA. Smokers are more often observed in RA patients, since smoking is associated with a higher incidence of RA [21,22,23]. Crowson et al., found that smoking, along with hypertension, have the highest population attributable risk (PAR), with non-smokers having a 23% lower risk compared to smokers. RA patients with the active disease have significantly lower high- and low-density lipoprotein cholesterol (HDL-c/LDL-c) levels compared to the healthy population [24]. Smoking was also significantly associated with a higher disease activity score, leading to worse clinical outcomes [25].

Hypertension and diabetes are also more prevalent in RA patients compared to healthy controls [24]. Treating patients with anti-rheumatics who also suffered from hypertension with medication resulted in a greatly reduced cardiovascular risk [21]. While hypertension is established as risk factor for cardiovascular disease, there is increasing evidence that systemic inflammation, as seen in RA patients, plays a central role in maintaining hypertension [26].

In addition to the traditional risk factors, patients with RA are more likely to have metabolic syndrome [27]. Metabolic syndrome is highly correlated with traditional risk factors such as hyperlipidemia, hypertension and diabetes mellitus and results in a relative risk for cardiovascular disease of 1.93 [28]. Higher disease activity or duration are also important, independent predictors of metabolic syndrome in RA patients [29]. Better weight control through dieting could improve this burden and improve some of the associated risks.

Many observational trials have shown that higher disease activity leads to increased cardiovascular risk, e.g., a Dutch study, where the researchers found that an increased DAS-28 score of 1 unit resulted in a 28% increased CV risk, irrespective of the disease duration [30]. Vice versa, one can expect a reduced CVD risk with lower disease activity, and in a large-scale trial done in 2015, Solomon et al., showed that a 10-point reduction in the CDAI (Clinical disease activity index) resulted in a 26% reduction in CV events [31]. 

## 4. Influence of Medication

### 4.1. csDMARDs

#### 4.1.1. Methotrexate

Methotrexate (MTX) is the anchor conventional synthetic disease-modifying anti-rheumatic drug (csDMARD) in the treatment of RA and reduced cardiovascular events by 20% in a recently updated meta-analysis that included nearly 200,000 RA patients [32].

The main mode of action of MTX in RA is the inhibition of the enzyme dihydrofolate reductase, which produces a key ingredient needed for DNA and RNA synthesis. This, in turn, causes a reduced amount of proliferated lymphocytes [33]. These proliferated lymphocytes excrete several key cytokines that are essential in maintaining the pro-inflammatory environment. By reducing these lymphocytes, the inflammation subsides and the patient experiences less pain and swelling. However, the mechanisms that lead to its cardio-protective effects have not yet been entirely elucidated. MTX seems to reduce TNF-α, IL-6 and C-reactive protein while also downregulating foam cell production [34,35]. The reduction of these pro-inflammatory molecules most likely signals the reduced systemic inflammation caused by MTX, which, in turn, leads to a reduced CV risk. 

The significant reduction in the CVD risk in patients with RA led to a randomized double-blinded placebo-controlled trial with low-dose MTX on general population patients that previously had a myocardial infarction or multi-vessel disease combined with either diabetes or metabolic syndrome [36]. Patients in the MTX arm received 15 mg once per week at the start that was increased to 20 mg once per week after 4 months. In contrast to the expectations, the investigators found no difference between the MTX and placebo arms with respect to the CVD endpoints. Possible explanations could be the relatively low dose of MTX and/or the low systemic inflammatory load in comparison to RA.

#### 4.1.2. Other csDMARDs

There have been relatively few studies on the CV risk of csDMARDs compared to MTX. A study done by our group showed a reduced risk for RA patients that used either methotrexate, sulfasalazine, or methotrexate, sulfasalazine and hydroxychloroquine [37]. Given the case–control design of the study, it was not optimally designed to give an effect estimation.

Antimalarial drugs such as hydroxychloroquine and chloroquine might have a positive effect on the reduction of CV events. In a retrospective study done by Sharma et al., a 72% decrease of CV events was seen [38]. Although, it must be noted that these results have not been replicated by other studies. A meta-analysis looking at hydroxychloroquine and chloroquine use in all rheumatic diseases found a significantly reduced CV risk of 28%. Possible causes of this reduced risk are reduced systemic inflammation and a reduction of the traditional risk factors such as hyperglycemia and hypercholesterolemia [39]. While there is not enough data to make a definitive conclusion, the first signs are positive.

Glucocorticoids such as prednisolone might increase the cardiovascular risk in RA patients in a dose- and duration-dependent manner. In a very large retrospective registry study, Ocon et al. showed that RA patients that used <5 mg daily or had a cumulative dose of <750 mg total had no increased CV risk [40]. Patients with higher daily doses, higher cumulative doses or longer treatment durations did show an increased CV risk. During the recent GLORIA trial, patients were given low-dose glucocorticoids (5 mg prednisolone) for 24 months. This resulted in an increased incidence of CV events of approximately 35% (2.4 cases per 100 patient years in the prednisolone group vs. 1.7 in the placebo group) [41].

Selective COX-2 inhibitors are known to increase the CV risk in the general population by approximately 35–40% compared to the placebo [42,43]. This increase is comparable to that of traditional NSAIDs such as ibuprofen or diclofenac [44]. There have not been any prospective studies investigating the CV risk of celecoxib or other selective COX-2 inhibitors specifically in RA patients. One could speculate that, on the one hand, this could be similar to the general population, while, on the other hand, in RA, NSAIDs might somewhat suppress inflammation and improve mobility, thus having favorable effects on the CV risk.

Studies investigating the effect of leflunomide on CV risk are rare. Two retrospective database cohort studies from the same research group showed a decreased relative risk for acute myocardial infarction and congestive heart failure in RA patients using leflunomide [45,46]. No prospective studies have been performed.

### 4.2. b- and tsDMARDs

During the last 20 years, numerous biological DMARDs (bDMARDs) have become available for RA treatment. Most of these bDMARDs specifically target proteins associated with inflammation, such as tumor necrosis factor (TNF) and interleukin 6 (IL-6), while others target the development of T and B cells (abatacept and rituximab, respectively). Newer targeted synthetic DMARDs, such as the Janus Kinase (JAK) inhibitors, affect the cytokine pathways of the immune system more specifically. As the fundamental research in the pathophysiology of atherosclerosis shows, the immune system has an important role in the development of atherosclerotic plaques [47]. TNF-α especially, but also IL-6, seem to contribute to endothelial dysfunction. Thus, it is hypothesized that treatment with medication inhibiting these proteins might reduce the risk of CVD in RA patients.

#### 4.2.1. TNF Inhibitors

TNF inhibitors specifically target TNF-α, a pro-inflammatory cytokine associated with the systemic inflammation in RA. TNF-a is produced by macrophages and foam cells during atherosclerotic plaque formation to maintain and progress plaque development [48,49]. Treatment with TNF inhibitors thus seems to impact both systemic inflammation and atherosclerotic plaque formation. Several studies have shown that the incidence of CVD in RA patients treated with these medications is reduced compared to patients without anti-inflammatory treatment or with csDMARDs [50,51,52,53,54]. A longer treatment duration with TNF inhibitors also seems to further reduce the CVD risk. Additionally, TNF inhibitors seem to increase HDL cholesterol in patients, which might contribute to its cardio-protective effects [55,56]. The decrease in systemic inflammation caused by TNF inhibitors also improves the HDL efflux capacity and reduces the risk of CVD independent of the HDL levels [14,57]. A proof-of-concept study also found a potential blood pressure-lowering effect [58]. However, these effects still need to be confirmed in prospective-controlled studies.

#### 4.2.2. Interleukin Inhibitors

Interleukin inhibitors used in RA treatment primarily focus on IL-6, another pro-inflammatory cytokine involved in RA pathology. Like TNF-a, its production is upregulated during the early stages of atherosclerotic plaque formation [48]. IL-6 inhibition results in a decrease in inflammation in most RA patients, but an increase in LDL cholesterol is also often measured. While this seems paradoxical, it still results in a decreased CVD risk comparable with TNF inhibition, partly because of an improvement in the HDL efflux capacity [59,60]. An IL-6 blockade might also reduce the lipoprotein(a) levels [61]. The decrease in inflammation thus outweighs the increase in LDL cholesterol. IL-6 inhibition also has a direct effect on the endothelium, improving endothelium function in high-risk RA patients [62]. This effect was also replicated in non-RA patients with atherosclerosis, in whom treatment with an IL-6 inhibitor resulted in reduced CV events and mortality [63].

#### 4.2.3. Abatacept

Abatacept reduces the inflammation in RA patients by inhibiting the activation of T cells, which restricts its ability to produce an immune response. Several studies indicated that abatacept might be more effective in decreasing cardiovascular risk in RA patients than csDMARDs and TNF inhibitors [64,65]. Abatacept also seems to have favorable effects on the insulin sensitivity, especially in diabetes patients [66]. Hypertension has also been described as a possible side effect of 1–10% [67]. However, the CVD reduction due to the anti-inflammatory effect of abatacept seems to not be affected [68,69].

#### 4.2.4. Rituximab

Rituximab is a CD-20 monoclonal antibody that reduces the B-cell count in patients. It increases the HDL cholesterol and improves endothelial function and arterial stiffness [70,71]. While its overall effect on the incidence of cardiovascular diseases is still unclear, myocardial ischemia and infarction have both been described as possible rare adverse events in rituximab users during infusion, although only in a few case reports [72]. It has yet to be elucidated whether its positive effect on the risk factors outweighs the possible CV side effects.

#### 4.2.5. JAK Inhibitors

JAK inhibitors are new targeted synthetic DMARDs used in treatment for RA that reduce inflammation by inhibiting the JAK-STAT pathway to decrease the production of several pro-inflammatory cytokines. The JAK-STAT pathway has a function in atherosclerotic CVD; however, more long-term studies are required to understand JAK inhibitor effects on the CVD risk [73,74]. The reported side effects of JAK inhibitors include hypercholesterolemia and thromboembolic events, such as deep vein thrombosis (DVT) and pulmonary embolism (PE) [75]. Caution is advised in patients with preexisting risk factors for thromboembolic events, such as higher age, obesity, smoking, history of DVT/PE or long periods of immobilization [76]. In several trials, the JAK inhibitors did not show an increased CVD risk compared to the placebo, cs-/bDMARDs or other JAK inhibitors [77,78]. However, more recently, the FDA issued a safety warning concerning the results of the “Oral surveillance” safety trial [79]. In this trial, patients were randomized to either tofacitinib 5 mg or 10 mg twice daily or a TNF inhibitor. Other inclusion criteria were ages over 50 and one or more cardiovascular risk factors [80]. During the trial, an increased risk of pulmonary embolism was found in the 10 mg group, and therefore, the dose was reduced to 5 mg twice daily during the remainder of the trial [81]. The incidence of MACE in the tofacitinib 5 mg group was 0.91 per 100 patient years vs. 0.73 per 100 patient years in the TNF inhibitor group, resulting in a hazard ratio of 1.33 (CI 95% 0.91–1.94). Non-inferiority was not proven, as the upper boundary of the confidence interval of the hazard ratio was higher than 1.8, indicating a higher CVD risk with the uses of tofacitinib in comparison to the TNF inhibitor. Another recent study suggested no significant differences in MACE and thrombosis incidence after 60 days of treatment between tofacitinib and etanercept. However, the number of studied patients, length of follow-up and number of arterial and venous thrombotic events were too low to reach valid conclusions [82]. The European Medicines Agency has also recommended that patients over 65 years of age, patients who are current or past smokers, patients with other CV risk factors and patients with other malignancy risk factors should only use tofacitinib if no other treatment is available [83].

## 5. Cardiovascular Risk Management

The increased cardiovascular risk in RA patients has been acknowledged by the current guidelines and has been implemented in several risk calculators [84]. The European League Against Rheumatism (EULAR) in their most recent guideline recommends screening every patient with RA at least every five years and more often if an increased risk was previously found [85]. Screening should be performed according to the national guidelines. For a 10-year CVD risk prediction, the use of a SCORE model is recommended. Calculated risk scores should be multiplied by 1.5 if the algorithm does not already account for RA as an independent risk factor. The EULAR recommends using the same treatment targets for the lipid levels and blood pressure as for the general population, with an additional emphasis on lifestyle interventions, and advises caution with prolonged NSAIDs and glucocorticoids usage. The European Society of Cardiology (ESC) also acknowledges the increased risk in RA but is less firm in its recommendations and suggests a low threshold for the assessment of the total CVD risk in adult patients and multiplying the calculated risk based on the disease activity by 1.5 [86]. The ESC also advises treating the CVD risk with similar interventions as for the general high-risk population.

While some well-known cardiovascular risk calculators, such as Framingham, have yet to include RA as a risk factor, several others have heeded these recommendations. The SCORE calculator includes a 1.5 multiplication factor for RA patients, and the most recent QRISK calculator multiplies the risk in RA patients by around 1.2. However, these calculators still seem to underestimate the risk in many RA patients, especially those categorized as low and intermediate risk. Only a small proportion of patients gets relocated to a more appropriate risk category by utilizing these multiplication factors. To correct this underestimation, an RA-specific calculator for CV risk screening was in development by the ATACC-RA group; however, the model could not be validated [87].

Despite such explicit advice and the availability of tools incorporating the increased cardiovascular risk of RA patients, screening in daily clinical practice is generally poorly done. RA patients often have undiagnosed or untreated risk factors, such as hypertension or hypercholesterolemia [88]. A study surveying general practitioners (GPs) about the cardiovascular risk of RA patients found that the majority did not recognize RA as an independent risk factor for cardiovascular diseases and did not perform the recommended screening assessments on RA patients in their practice [89]. They also found that only a small minority calculated cardiovascular risk scores using the 1.5 multiplication factor. Even after screening, when general practitioners and internists received notice of risk stratification, a more recent study found that only one-third of patients with an indication of treatment received this [90]. 

## 6. Conclusions

Even nowadays, RA patients still have an increased risk of developing CVD, due to both an increased prevalence of “traditional” CV risk factors, as well as systemic inflammation. Advances in anti-inflammatory treatment partly mitigate this risk by reducing the inflammation. Currently, screening (and treatment, if indicated) for CV risk factors is still insufficient, resulting in undiagnosed and untreated risk factors. RA patients need optimal control of their systemic inflammation and mandatory regular screening for CV risk factors to turn their CVD risk towards that of the general population.

## Data Availability

Not applicable.
